# Peptidomic Analysis of Skin Secretions of the Caribbean Frogs *Leptodactylus insularum* and *Leptodactylus nesiotus* (Leptodactylidae) Identifies an Ocellatin with Broad Spectrum Antimicrobial Activity

**DOI:** 10.3390/antibiotics9100718

**Published:** 2020-10-20

**Authors:** Gervonne Barran, Jolanta Kolodziejek, Laurent Coquet, Jérôme Leprince, Thierry Jouenne, Norbert Nowotny, J. Michael Conlon, Milena Mechkarska

**Affiliations:** 1 Department of Life Sciences, Faculty of Science and Technology, The University of The West Indies, St. Augustine Campus, Trinidad and Tobago; brandon-barran@hotmail.com; 2 Viral Zoonoses, Emerging and Vector-Borne Infections Group, Department of Pathobiology, Institute of Virology, University of Veterinary Medicine, Veterinärplatz 1, A-1210 Vienna, Austria; Jolanta.Kolodziejek@vetmeduni.ac.at (J.K.); Norbert.Nowotny@vetmeduni.ac.at (N.N.); 3 CNRS UMR 6270, PISSARO, Institute for Research and Innovation in Biomedicine (IRIB), Normandy University, 76000 Rouen, France; laurent.coquet@univ-rouen.fr (L.C.); thierry.jouenne@univ-rouen.fr (T.J.); 4 Inserm U1239, PRIMACEN, Institute for Research and Innovation in Biomedicine (IRIB), Normandy University, 76000 Rouen, France; jerome.leprince@univ-rouen.fr; 5 Department of Basic Medical Sciences, College of Medicine, Mohammed Bin Rashid University of Medicine and Health Sciences, Dubai Helathcare City, P.O. Box 505055, Dubai, UAE; 6 Diabetes Research Group, School of Biomedical Sciences, University of Ulster, Coleraine BT52 1SA, Northern Ireland, UK

**Keywords:** antibiotic resistance, antimicrobial peptides, frog skin secretions, hemolysis, *Leptodactylus*, norepinephrine stimulation, ocellatins, peptidomic analysis, phylogenetics, Trinidad

## Abstract

Ocellatins are peptides produced in the skins of frogs belonging to the genus *Leptodactylus* that generally display weak antimicrobial activity against Gram-negative bacteria only. Peptidomic analysis of norepinephrine-stimulated skin secretions from *Leptodactylus insularum* Barbour 1906 and *Leptodactylus nesiotus* Heyer 1994, collected in the Icacos Peninsula, Trinidad, led to the purification and structural characterization of five ocellatin-related peptides from *L. insularum* (ocellatin-1I together with its (1–16) fragment, ocellatin-2I and its (1–16) fragment, and ocellatin-3I) and four ocellatins from *L. nesiotus* (ocellatin-1N, -2N, -3N, and -4N). While ocellatins-1I, -2I, and -1N showed a typically low antimicrobial potency against Gram-negative bacteria, ocellatin-3N (GIFDVLKNLAKGVITSLAS.NH_2_) was active against an antibiotic-resistant strain of *Klebsiella pneumoniae* and reference strains of *Escherichia coli*, *K. pneumoniae*, *Pseudomonas aeruginosa*, and *Salmonella typhimurium* (minimum inhibitory concentrations (MICs) in the range 31.25–62.5 μM), and was the only peptide active against Gram-positive *Staphylococcus aureus* (MIC = 31.25 μM) and *Enterococcus faecium* (MIC = 62.5 μM). The therapeutic potential of ocellatin-3N is limited by its moderate hemolytic activity (LC_50_ = 98 μM) against mouse erythrocytes. The peptide represents a template for the design of long-acting, non-toxic, and broad-spectrum antimicrobial agents for targeting multidrug-resistant pathogens.

## 1. Introduction

In an ongoing effort to address the global problem of antimicrobial resistance (AMR) and to promote the research and development of new antibiotics against emerging multidrug-resistant bacteria, in 2017, the World Health Organization published a list of “priority pathogens” that cause increased morbidity and mortality and greatly impact healthcare costs [1]. Infections caused by antibiotic-resistant Gram-negative pathogens, such as extended-spectrum β-lactamase (ESBL) *Escherichia coli* and multidrug-resistant *Acinetobacter baumannii*, *Pseudomonas aeruginosa*, and *Klebsiella pneumoniae*, are of particular concern because the currently available treatment options are often ineffective and there are only a few antimicrobial drugs in the pipeline [2]. In order to address this crisis, the AMR Action Fund, encompassing more than 20 leading biopharmaceutical companies, was launched in July 2020, with the aim of bringing at least four new treatments to patients by 2030 [3]. AMR has been described as “a slow tsunami that threatens to undo a century of medical progress” [4]. Consequently, there is clearly a need to identify and develop new kinds of antimicrobial drugs with acceptable toxicological and pharmacokinetic properties that may be used to treat infections caused by multidrug-resistant pathogenic microorganisms. Anti-infective compounds based upon peptides are one such alternative to conventional antibiotics due to their rapid and non-specific mode of action, as well as their abilities to inhibit biofilm formation and act synergistically with established antibiotics [5].

Several frog species produce peptides with broad-spectrum antibacterial and antifungal activities that are also able to disrupt the plasma membrane of mammalian cells and more than 1000 such peptides have been described (reviewed in [6,7,8,9,10,11]). Although their precise biological role is incompletely understood, these peptides probably function as a component of the animal’s system of innate immunity, playing a role in the first-line defense against invading pathogens [12,13]. As peptides from different frog species may contain regions of structural similarity, they can be grouped into families for the purpose of classification. In addition, several of these peptides stimulate the production of pro- or anti-inflammatory cytokines by macrophages, inhibit viral replication, exert cytotoxicity towards cancer cells, and stimulate insulin release [14]. Consequently, it is more informative to refer to them as host-defence peptides (HDPs) than exclusively as antimicrobial peptides. Typically, each frog species has a “finger-print” arsenal of HDPs belonging to different families, whose primary structures can be used as a tool for elucidating the evolutionary history and complex phylogenetic relations of the different frog genera [15,16,17].

The genus *Leptodactylus*, or nest-building frogs, currently contains 78 species distributed from southern North America to central South America, including the West Indies [18]. Several frogs belonging to this genus have been shown to release structurally-related HDPs with antimicrobial activity into their skin secretions: *Leptodactylus ocellatus* [19,20,21], *Leptodactylus fallax* [22], *Leptodactylus pentadactylus* [23,24], *Leptodactylus laticeps* [25,26], *Leptodactylus syphax* [27], *Leptodactylus validus* [28], *Leptodactylus labyrinthicus* [29,30], *Leptodactylus pustulatus* [31], *Leptodactylus latrans* [32], and *Leptodactylus vastus* [33]. These peptides were initially named after the frog species from which they were first isolated, but they are now classified as ocellatins according to a generally accepted nomenclature [34]. In addition to the ocellatins, conformationally flexible glycine/leucine-rich plasticins, which lack antimicrobial activity, have been isolated from the skin secretions of *L. pentadactylus* [24] and *L. laticeps* [26].

As part of a program of investigation to systematically examine the frog species of Trinidad and Tobago for the presence of dermal biologically-active HDPs, this study involves an investigation of two *Leptodactylus* species that have not been studied previously: *Leptodactylus insularum* Barbour 1906 and *Leptodactylus nesiotus* Heyer 1994. The San Miguel Island frog *L. insularum* (also known as Barbour’s Thin-Toed frog) is a medium to large frog (snout-vent-length (SVL) for sub-adults/adults is in the range of 51–83 mm). Previously classified as *L. pentadactylus* and *Leptodactylus bolivianus* [35], *L. insularum* is found from Costa Rica to Trinidad, but it has not been recorded in Tobago. In Trinidad, this species has been found at low elevations in the central and southeast parts of the island. The preferred habitats of the frog include forested areas and swamp margins. Because of its wide distribution range, tolerance of varied habitats, and presumably large populations, *L. insularum* is classified by the International Union for Conservation and Nature (IUNC) Red List of Threatened Species as a species of Least Concern. The Trinidad Thin-Toed frog *L. nesiotus* is a small terrestrial frog (adult males SVL 33 mm and sub-adults SVL 21–28 mm). This species was considered to be endemic to Trinidad until Jairam and Fouquet [36] reported, for the first time, its presence on the South American continent in Guyana, Suriname, and French Guiana. *L. nesiotus* can be found inhabiting open and swampy areas of the Icacos Peninsula, as well as many scattered locations in southern Trinidad [35]. *L. nesiotus* is listed as a Vulnerable Species by the IUCN.

Both frog species investigated in this study were identified on the basis of their audio calls and characteristic morphologym as described by Murphy et al. [35]. An injection of norepinephrine was used to stimulate the release of HDPs into skin secretions, which were purified and characterized by reversed-phase HPLC in combination with MALDI-TOF mass spectrometry and automated Edman degradation. The peptides isolated from *L. insularum* are denoted by I and those isolated from *L. nesiotus* by N. Peptides from paralogous genes are differentiated by numerals, e.g., ocellatin-1N and ocellatin-2N. The antimicrobial potencies of synthetic replicates of four of the ocellatins against a range of Gram-positive and Gram-negative reference bacteria, including antibiotic-resistant strains, as well as their cytotoxic activities against mouse erythrocytes, were determined. Additionally, the amino acid sequences of the peptides were used in cladistic analyses to gain insight into phylogenetic relationships among the *Leptodactylus* species studied to-date.

## 2. Results

### 2.1. Purification of the Peptides from L. insularum

**Figure 1:**
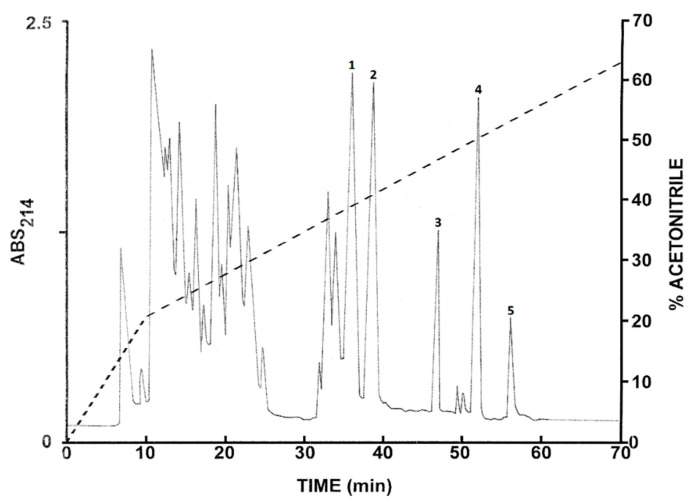
Elution profile on a semipreparative Vydac C-18 column of pooled skin secretions from nine *Leptodactylus insularum* frogs after prior concentration on Sep-Pak cartridges. The dashed line shows the concentration of acetonitrile in the eluting solvent. Peaks 1–5 contain ocellatin-related peptides that were purified to near homogeneity by further chromatography on Vydac C-8 and dimethylphenyl columns. Absorbance was monitored at 214 nm and fractions (1 min) were collected.

The pooled skin secretions from *L. insularum* frogs were initially concentrated by passage through Sep-Pak C-18 cartridges, followed by chromatography on a Vydac C-18 semipreparative reversed-phase HPLC column (Figure 1). Further purification to near homogeneity of the peptides at the prominent peaks (1–5) was achieved by successive chromatographies on semipreparative Vydac C-4 and Vydac dimethylphenyl columns (chromatograms not shown). Subsequent structural analysis revealed that peak 1 contained the 1–16 fragment of ocellatin-1I (ocellatin-1I (1–16)), peak 2 the 1-16 fragment of ocellatin-2I (ocellatin-2I (1–16)), peak 3 ocellatin-1I, peak 4 ocellatin-2I, and peak 5 ocellatin-3I. All peptides used in the antimicrobial assays displayed a symmetrical peak shape and their purity was estimated to be >98% by MALDI-TOF mass spectrometry.

### 2.2. Purification of the Peptides from L. nesiotus

**Figure 2:**
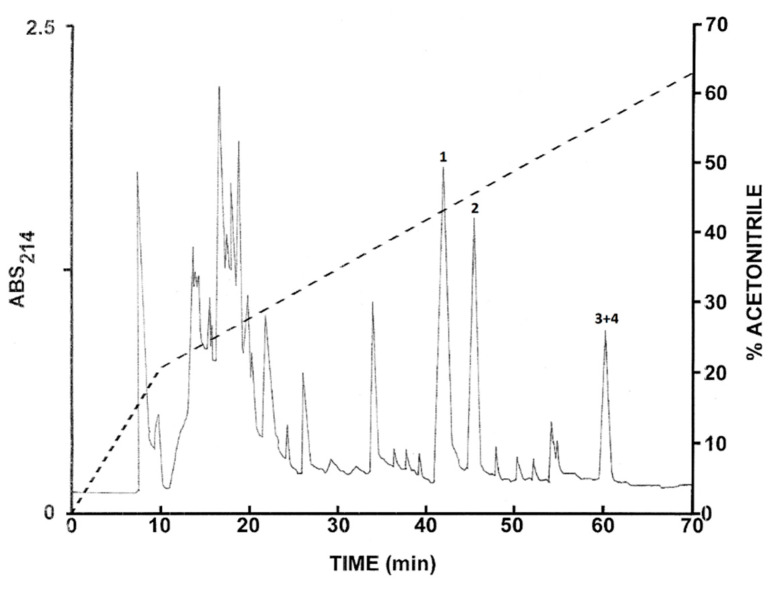
Elution profile on a semipreparative Vydac C-18 column of pooled skin secretions from seven *Leptodactylus nesiotus* frogs after prior concentration on Sep-Pak cartridges. The dashed line shows the concentration of acetonitrile in the eluting solvent. Peaks 1–4 contain ocellatins that were purified to near homogeneity by further chromatography on Vydac C-8 and dimethylphenyl columns. Absorbance was monitored at 214 nm.

The pooled skin secretions from *L. nesiotus* were subjected to the same chromatographic procedures used to purify peptides from *L. insularum.* The elution profile on a Vydac C-18 semipreparative column is shown in Figure 2 and the peptides of major abundance at peaks 1–4 were collected. Subsequent structural analysis revealed that peak 1 contained ocellatin-1N, peak 2 ocellatin-2N, and peak 3 and 4 ocellatin-3N and ocellatin-4N. The final purity of the peptides was estimated to be >98%.

### 2.3. Structural Characterization

**Figure 3:**
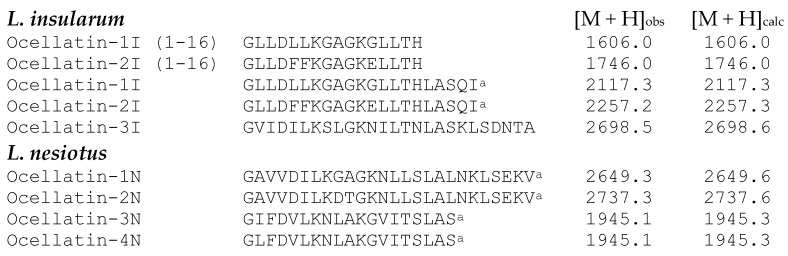
Amino acid sequences and molecular masses of the peptides isolated from norepinephrine-stimulated skin secretions from *L. insularum* and *L. nesiotus*. [M + H]_obs_ denotes the observed molecular mass and [M + H]_calc_ denotes the mass calculated from the proposed structures. ^a^ Denotes C-terminal α-amidation. (1–16) Indicates a truncated peptide.

The amino acid sequences of the ocellatins isolated from *L. insularum* and *L. nesiotus* skin secretions were established without ambiguity by automated Edman degradation (Figure 3).

The molecular masses of the peptides, determined by MALDI-TOF mass spectrometry, were consistent with the proposed structures and demonstrated that ocellatin-1I and -2I from *L. insularum* and all ocellatin peptides from *L. nesiotus* were C-terminally α-amidated. Ocellatin-1I and -2I differ by three amino acid residues (positions 5, 6, and 12). In addition, (1-16) fragments of both ocellatin-1I and -2I were purified from the *L. insularum* secretions. Ocellatin-1N and -2N differ by two amino acid residues at positions 9 and 10, whereas ocellatin-3N and -4N differ only by the substitution of Ile by Leu at position 2. The physicochemical properties of the ocellatin-related peptides isolated in this study are shown in Table 1. The Grand Average of Hydropathy (GRAVY) was determined using the hydrophobicity scales of Kyte and Doolittle [37]. The presence of helical domains in the peptides was predicted using the AGADIR program [38]. The isoelectric point (pI) was calculated using the following web-site: http://www.bachem.com/service-support/peptide-calculator/.

### 2.4. Antimicrobial and Hemolytic Activities

The minimum inhibitory concentration (MIC) values for synthetic ocellatin-1I and -2I and ocellatin-1N and -3N against strains of Gram-positive bacteria *Staphylococcus aureus, Enterococcus faecium*, and *Enterococcus faecalis* and Gram-negative bacteria *Escherichia coli*, *Klebsiella pneumoniae*, *Pseudomonas aeruginosa,* and *Salmonella typhimurium*, as well as ampicillin-resistant *S. aureus* and antibiotic-resistant *K. pneumoniae*, are shown in Table 2. Ocellatin-1I, -2I, and -1N exhibited relatively weak antimicrobial activities (MICs in the range of 62.5–250 µM) against the Gram-negative bacteria only. Ocellatin-3N from *L. nesiotus* displayed the greatest activity against all Gram-positive and Gram-negative microorganisms tested, including the multidrug-resistant strains (MICs in the range of 31.25–62.5 μM). However, this peptide was 4–8 times less potent against the Gram-positive bacterium *E. faecalis* (MIC = 250 μM).

Antibiotic sensitivities towards ampicillin (AMP), vancomycin (VAN), and ciprofloxacin (CIP) of all bacterial strains used were determined prior to setting up the experiments so that an appropriate control could be used in parallel with incubation with the peptides. The MICs for the antibiotics were as follows: *E. faecalis* AMP 4 μg/mL (VAN 64 µg/mL and CIP > 64 µg/mL)*; E. coli* AMP 6.25 μg/mL; *K. pneumoniae* CIP 0.13 μg/mL; *P. aeruginosa* CIP 0.25 μg/mL (AMP 512 μg/mL and VAN > 512 μg/mL); *S. typhimurium* CIP 0.13 μg/mL; and *S. aureus* (ATCC BAA-2312) VAN 2 μg/mL and AMP 16 μg/mL. The antibiotic-resistant *K. pneumoniae* strain (ATCC BAA-2814) was not responsive to any of the antibiotics tested in concentrations ≤ 512 μg/mL. Similarly, *E. faecium* (ATCC 19434) was not inhibited by either AMP or VAN in concentrations ≤ 512 μg/mL, as well as by CIP ≤ 64 μg/mL. The *S. aureus* strain (ATCC 12600) showed a high resistance to AMP (MIC = 100 μg/mL), but was sensitive to VAN (MIC = 2 μg/mL).

When tested for cytotoxicity against mouse erythrocytes in vitro, both ocellatin-1I and -2I were weakly hemolytic (LC_50_ > 250 µg/mL). Ocellatin-1N was non-hemolytic (LC_50_ > 500 µg/mL), whereas ocellatin-3N was the most cytotoxic (LC_50_ = 210 µg/mL, equivalent to 98 μM) out of the four peptides tested.

### 2.5. Cladistic Analysis

**Figure 4:**
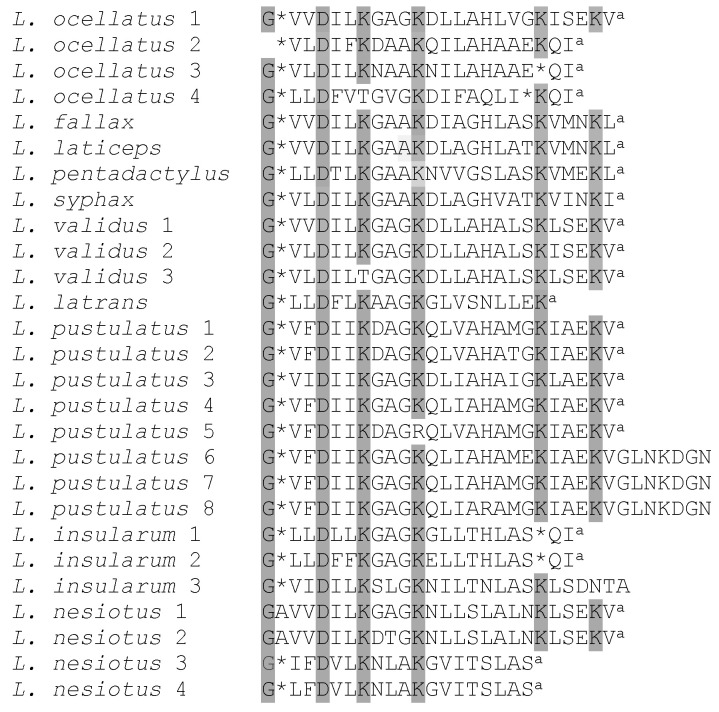
Sequence alignment of the ocellatin peptides from *L. insularum* and *L. nesiotus* with the orthologous peptides from other *Leptodactylus* species isolated to-date. Gaps denoted by (*) are introduced into certain sequences in order to maximize the structural similarity of the peptides. Shading is used to indicate the amino acid residues that have been very strongly conserved. ^a^ Denotes C-terminal α-amidation.

**Figure 5:**
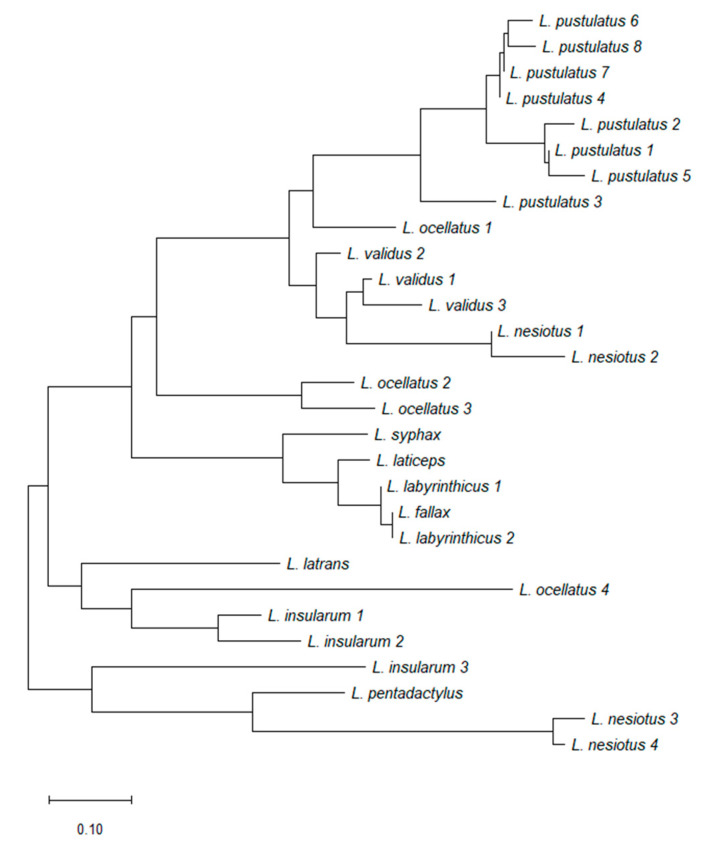
A phylogenetic tree generated using the neighbor-joining method with Poisson correction based upon the primary structures of the ocellatin peptides isolated from frogs belonging to the genus *Leptodactylus* that are shown in Figure 4.

Figure 4 shows the primary structures of 27 ocellatins isolated from 10 *Leptodactylus* species. These amino acid sequences were used to construct the optimal phylogenetic tree shown in Figure 5. It can be seen that evolutionary pressure was added, primarily to conserve the charged residues (Lys and Asp) in this family of peptides. The tree was drawn to scale, with branch lengths being given in the same units as those of the evolutionary distances used to infer the phylogenetic tree.

## 3. Discussion

This study has described the purification of five peptides from norepinephrine-stimulated skin secretions of *L. insularum* and four peptides from skin secretions of *L. nesiotus*. Determination of the primary structures of the peptides revealed that they represent orthologues of the ocellatin frog skin peptides named after the first member to be identified from a skin secretion of *L. ocellatus* [19]. The peptidomic approach (reversed-phase HPLC combined with MALDI-TOF mass spectrometry and automated Edman degradation) allowed the characterization of all of the peptides that were present in major abundance in the secretions. The advantage of the peptidomic approach is that those peptides with low or no antimicrobial activity would have been easily missed if the more traditional bioassay-guided method was utilized for identification of the HDPs [9].

Skin secretions of *L. insularum* contained two truncated (1–16) fragments of ocellatin-1I and -2I (Figure 3). The samples from this species were collected, handled, and processed in the author’s laboratory at the same time as those from *L. nesiotus*, but no such fragments were detected in the secretions from the latter. The presence of shorter ocellatin-related peptides appears to be a common feature for many of the *Leptodactylus* frogs studied to-date, irrespective of the method used for collection of the secretions [20,22,28,29]. It is unclear whether the ocellatin fragments originate from the proteolytic processing of full-length ocellatins by the peptidases that are present in skin secretions or are products of different genes.

Frog skin HDPs are associated with extreme structural hypervariability and several studies (reviewed in [15]) have shown that comparisons of their amino acid sequences have proved to be of value in elucidating the evolutionary history and phylogenetic relationships of frogs within a particular genus. Such analyses can be used in conjunction with those based upon comparisons of nucleotide sequences of orthologous genes and morphological criteria. Cladistic analysis based upon the primary structures of the ocellatins from *Leptodactylus* frogs supports the conclusion based upon morphological criteria [18] that *L. nesiotus* and *L. validus* are closely related phylogenetically, whereas *L. insularum* is most closely related to *L. ocellatus*.

It is well-documented that most of the ocellatins isolated to-date show low hemolytic activity and display only weak antimicrobial activity targeting preferentially Gram-negative bacteria [21,22,24,26,32]. However, ocellatin 4, isolated from *L. ocellatus*, displayed broad-spectrum antimicrobial activity by inhibiting both *E. coli* and *S. aureus* (MICs = 64 μM) [20] and it was also strongly hemolytic (LC_50_ = 14.3 μM). Ocellatin-related fragments may contribute to the general protection exerted by the full-length peptides, as some of them have been documented to possess various degrees of antimicrobial and hemolytic activities [27,33]. The in vitro antimicrobial and hemolytic activities of synthetic replicates of the ocellatins -1I, -2I, -1N, and -3N (selected on the basis of their higher hydrophobicities) were investigated using a range of reference bacteria, including drug-resistant bacterial strains, and mouse erythrocytes (Table 2). Ocellatin-3N isolated from skin secretions of *L. nesiotus* was the only peptide that displayed broad-spectrum antimicrobial activity (MICs = 31.25–62.5 μM) against Gram-positive and Gram-negative bacteria, including drug-resistant strains. The hemolytic activity of ocellatin-3N (LC_50_ = 98 μM) was appreciably greater than the corresponding activities of fallaxin (LC_50_ > 200 μM) [22], pentadactylin (LC_50_ > 400 μM) [23], and laticeptin (LC_50_ > 400 μM) [25]. However, the peptide, while displaying comparable activity against a range of Gram-positive and Gram-negative bacteria to that of ocellatin 4 [20], was nearly seven-fold less hemolytic. The cytotoxicity of ocellatin-3N against cell lines derived from human tissues, such as A549 alveolar basal epithelial cells and HUVEC umbilical vein endothelial cells, will be addressed in future studies. In addition, the activity of the peptide against reference strains and clinical isolates of opportunist yeast pathogens, such as *Candida albicans* and *Candida parapsilosis*, will be investigated.

**Figure 6:**
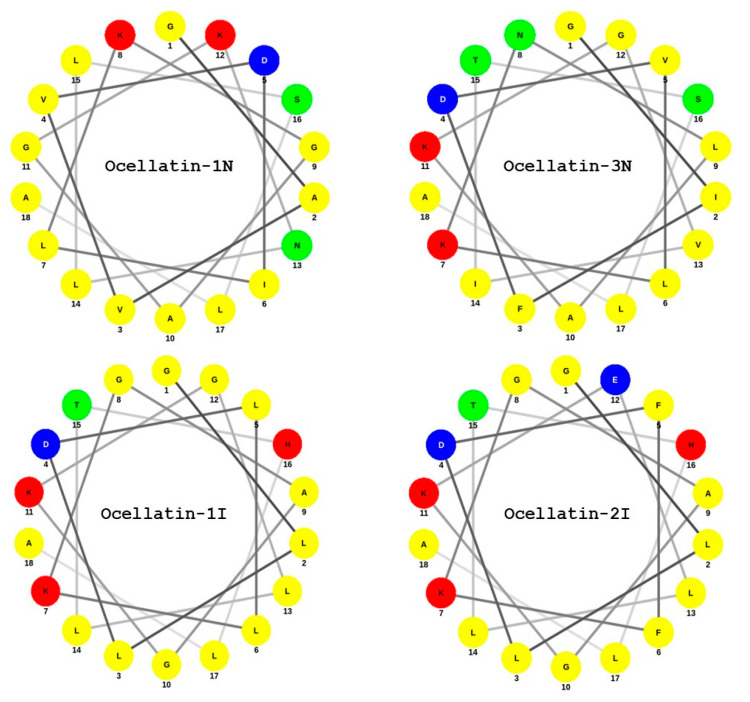
Schiffer–Edmundson wheel representation of the (1–18) regions of four of the ocellatins, isolated from *L. insularum* and *L*. *nesiotus*. Amino acid color code: Red—polar/basic (Lys, His); blue—polar/acid (Asp, Glu); green—polar/uncharged; and yellow—nonpolar/hydrophobic.

The factors that determine the activity of any antimicrobial peptide are the helicity, amphipathicity, hydrophobicity, and charge [39,40,41,42]. These parameters are strongly interrelated, which makes predictions based on the amino acid sequence of the peptide antimicrobial activity and mode of action difficult. Studies with model α-helical peptides have shown that to exert activity against Gram-positive bacteria, the peptides need to adopt stabilized amphipathic conformation, while that is not always needed in the case of Gram-negative bacteria [39]. Similarly, it has been demonstrated that the more amphipathic a peptide is, the more hemolytic it becomes for human erythrocytes [43]. As is the case for the vast majority of frog skin antimicrobial peptides, the ocellatins from *L. insularum* and *L. nesiotus* are cationic (charge at pH 7 between +1 and +3). In particular, ocellatin-3N has an overall cationic charge of +2, which could facilitate electrostatic interactions with the negatively charged bacterial cell membranes. Two regions of helicity were predicted for this peptide between amino acid residues 4–11 and 13–18 (Table 1). A Schiffer and Edmundson [44] wheel projectionof ocellatin-3N (Figure 6) demonstrated that the amphipathicity of the α-helix is high, with the polar Asp^4^, Lys^7^, and Lys^11^ residues aligning on one face of the helix and the hydrophobic Ile^2^, Leu^6^, Leu^9^, Val^13^, and Leu^17^ residues aligning on the opposite face. In addition, ocellatin-3N was the most hydrophobic of peptides tested in this study, with a GRAVY value of 0.911. It is suggested, therefore, that the observed potency of ocellatin-3N against Gram-positive bacteria and the hemolytic activity are a consequence of the combination of a high degree of amphipathicity and hydrophobicity of the peptide. Although ocellatin-1N is even more cationic than ocellatin-3N, its hydrophobicity is low. Similarly, Figure 6 demonstrates that the amphipathicities of ocellatin-1I and -2I are low, which accounts for the weak antimicrobial activity of these three peptides.

Bessa et al. [45] reported that ocellatins isolated from *L. pustulatus* acted preferentially on clinical isolates of multidrug-resistant *P. aeruginosa* (MICs 16–256 µg/mL) compared with reference strains (MICs > 520 µg/mL). In particular, ocellatin-PT3 displayed an ability to inhibit biofilm formation (concentrations 4–8 times higher than the MIC) and showed synergistic effects with the antibiotics ciprofloxacin and ceftazidime, so the peptide was proposed as a promising lead molecule for the design and development of novel therapeutic agents against drug-resistant *P. aeruginosa* biofilms.

However, the clinical utility of antimicrobial peptides is limited by their short half-life in circulation and their toxicities against human cells. Several strategies have been employed to increase the stability of HDPs, including the substitution of amino acid residues by D-isomers and unnatural amino acids; modification of the peptide termini; dimerization and multimerization of the peptide; cyclization; conjugation with polymers, sugars, and albumin; and the use of peptidase inhibitors [46]. Similarly, the cationicity, hydrophobicity, and amphipathicity of HDPs may be selectively manipulated to increase their antimicrobial potency, while simultaneously reducing their cytotoxicity to mammalian cells [41]. Moreover, the use of suitable carriers and optimized delivery systems, such as inorganic materials, polymers, and self-assembly lipid-based and nanomaterial-based structures, has been reported for both the systemic and local application of AMPs (reviewed in [47,48]). Such approaches would be expected to increase AMP stability, controlled release, and reduced toxicity and simultaneously minimize the potential side effects and/or overcome undesired host immune responses, thus alleviating many of the challenges that AMPs face as therapeutic agents. In this light, we propose future structure–activity studies that will involve the synthesis of long-acting analogs of ocellatin-3N with increased broad-spectrum activity against multidrug-resistant clinical isolates of pathogenic microorganisms and the ability to inhibit biofilm formation.

## 4. Materials and Methods

### 4.1. Collection of Skin Secretions

Relevant permits approving the collection and sampling of live animals were granted by the Wildlife Section, Forestry Division, Trinidad (Special Game License with nationwide validity was issued on 21 June 2016) and by the University of the West Indies (UWI) Campus Ethics Committee (CEC234/07/16). Adult and sub-adult *L. insularum* frogs (*n* = 9; SVL 55–85 mm; weight 13.7–74.9 g; sex not determined) and sub-adult specimens of *L. nesiotus* (*n* = 7; SVL 21–28 mm; weight 1.1–1.9 g; sex not determined) were collected at 10°4′49″ N, 61°53′25″ W in Cedros (Icacos, Trinidad) in May 2019. Species identification was based on audible calls and visual characteristics [35]. The animals were taken to a nearby base for the collection of skin secretions, which was carried out by authorized investigators. The *L. insularum* frogs were injected via the dorsal lymph sac with norepinephrine hydrochloride (NE; 40 nmol/g body weight), as previously described [49]. The much smaller *L. nesiotus* frogs were immersed in distilled water (130 mL) containing 40 nmol/g body weight NE for 15 min, as described [49]. The collection solutions were acidified by the addition of concentrated hydrochloric acid (final concentration 1%, *v*/*v*) and immediately frozen for transfer to Ulster University. The frogs were monitored closely over a period of a few hours for any signs of distress and were subsequently released unharmed at the site of collection.

### 4.2. Purification of the Peptides

The solutions containing the secretions from each animal were pooled separately and concentrated by passage at a flow rate of approximately 2 mL/min through nine (*L. insularum*) and three (*L. nesiotus*) Sep-Pak C-18 cartridges (Waters Associates, Milford, MA, USA) connected in series. Bound material was eluted with acetonitrile/water/trifluoroacetic acid (TFA) (70.0:29.9:0.1, *v*/*v*/*v*) and freeze-dried. The material was redissolved in 0.1% (*v*/*v*) TFA/water (2 mL) and injected onto a semipreparative (1.0 cm × 25 cm) Vydac 218TP510 (C-18) reversed-phase HPLC column (Grace, Deerfield, IL, USA) equilibrated with 0.1% (*v*/*v*) TFA/water at a flow rate of 2 mL/min. The following linear gradients were used to elute the peptides: 0% to 21% (*v*/*v*) acetonitrile over 10 min, followed by 21% to 63% (*v*/*v*) over 60 min. Absorbance was monitored at 214 nm and fractions (1 min) were collected using a BioRad 2110 fraction collector.

The peaks designated 1–5 in Figure 1 and 1–4 in Figure 2 were shown to contain components with molecular masses in the range of 1–4 kDa by MALDI-TOF mass spectrometry. These peptides were purified to near homogeneity by successive chromatographies on (1.0 cm × 25 cm) Vydac 214TP510 (C-4), (1.0 cm × 25 cm) Vydac 219TP510 (dimethylphenyl), and (1.0 cm × 25 cm) Vydac 208TP510 (C-8) columns. For the more hydrophilic peptides (retention times < 35 min), the concentration of acetonitrile in the eluting solvent was raised from 14% to 42% (*v*/*v*) over 50 min and for the more hydrophobic peptides, from 21% to 56% (*v*/*v*) over 50 min. The flow rate was 2 mL/min.

### 4.3. Structural Characterization

The monoisotopic molecular masses of the purified ocellatins were determined by MALDI-TOF mass spectrometry using an UltrafleXtreme instrument (Bruker Daltonik, Bremen, Germany). Full details of the procedure, including calibration of the instrument with peptides of a known molecular mass in the 1–4 kDa range, have been provided [50,51]. The accuracy of mass determinations was <0.02%. The primary structures of the purified peptides were determined by automated Edman degradation using an Applied Biosystems model 494 Procise sequenator (Foster City, CA, USA).

### 4.4. Synthetic Peptides

The four ocellatin peptides used in this study for an evaluation of antimicrobial and hemolytic activities were supplied in crude form by EZBiolab Inc. (Carmel, IN, USA). The peptides were purified by reversed-phase HPLC on a (2.2 cm × 25 cm) Vydac 218TP1022 (C-18) column equilibrated with acetonitrile/water/TFA (35.0/64.9.9/0.1, *v*/*v*/*v*) at a flow rate of 6 mL/min. The concentration of acetonitrile was raised to 63% (*v*/*v*) over 60 min using a linear gradient. Absorbance was measured at 214 nm and the major peak in the chromatogram was collected by hand. The identities of the peptides were confirmed by electrospray mass spectrometry and their final purities were estimated to be >98%.

### 4.5. Antimicrobial Assays

Minimum inhibitory concentrations (MICs) were determined in duplicate by a standard double dilution method according to CSLI guidelines [52] using 96-well microtiter cell-culture plates, as previously described [49]. All reference strain bacteria, including *S. aureus* (ATCC 12600), *E. faecium* (ATCC 19434), *E. faecalis* (ATCC 51299), *E. coli* (ATCC 35218), *K. pneumoniae* (ATCC 49472), *P. aeruginosa* (ATCC 27853), and *S. typhimurium* (ATCC 14028), as well as *S. aureus* (ATCC BAA-2312) and antibiotic-resistant *K. pneumoniae* (ATCC BAA-2814), were obtained from the Microbiology Research Group at the Department of Life Sciences (DLS), Faculty of Science and Technology (FST), University of the West Indies. Control incubations were carried out in parallel with increasing concentrations of antibiotics (ampicillin for *S. aureus, E. faecalis*, and *E. coli*; vancomycin for *S. aureus* (ATCC BAA-2312); and ciprofloxacin for the sensitive *K. pneumoniae* strain, *P. aeruginosa*, and *S. typhimurium*), in order to monitor the validity and reproducibility of the assays. The published antibiotic sensitivity/resistance profiles for all bacterial strains were confirmed in the authors’ laboratory prior to setting up the MIC experiments.

### 4.6. Hemolysis Assay

All procedures involving mice were approved by Ulster University (UU) Animal Ethics Review Committee and were carried out in accordance with the UK Animals (Scientific Procedures) Act 1986 and EU Directive 2010/63EU for animal experiments. Hemolytic activity against erythrocytes from NIH Swiss female mice was determined as previously described [22]. Control incubations were carried out in parallel in Krebs Ringer Bicarbonate buffer only or with 1% v/v Triton-X100, in order to determine the absorbance associated with 0% and 100% hemolysis, respectively. The LC_50_ value was taken as the mean concentration of peptide producing 50% hemolysis in three independent experiments.

### 4.7. Cladistic Analysis

The optimum phylogenetic tree was constructed using the neighbor-joining method [53]. The evolutionary distances were computed using the Poisson correction method [54] and are given as the number of amino acid substitutions per site. All positions containing alignment gaps and missing amino acid residues were only eliminated in pairwise sequence comparisons (pairwise deletion option). Phylogenetic analyses were conducted in MEGA X [55].

## 5. Conclusions

In contrast to the majority of previously isolated ocellatin-related peptides with well-documented weak antimicrobial activity towards Gram-negative bacteria only, ocellatin-3N from skin secretions of the Caribbean frog *L. nesiotus* displays broad spectrum activity against a range of Gram-negative and Gram-positive bacteria, including antibiotic-resistant strains. Consequently, this peptide could serve as a template for development into an antimicrobial agent with an improved potency and reduced cytotoxicity for red blood cells. In addition, this study has contributed to our understanding of amphibian biodiversity in Trinidad and the primary structures of the newly characterized ocellatin-related peptides provide insight into the phylogenetic relationships amongst Leptodactylid frogs.

## Figures and Tables

**Table 1:** Physicochemical properties of the ocellatin-related peptides isolated in this study from norepinephrine-stimulated skin secretions of *L. insularum* and *L. nesiotus*.

Peptide	No. of Amino Acids	Charge at pH 7	pI	GRAVY	Alpha-Helical Domain *
Ocellatin-1I (1–16)	16	+1	9.9	0.488	2–9
Ocellatin-2I (1–16)	16	0	7.8	0.169	none
Ocellatin-1I	21	+2	10.6	0.648	2–9, 13–19
Ocellatin-2I	21	+1	9.9	0.405	11–19
Ocellatin-3I	26	+1	9.8	0.205	2–10, 13–21
Ocellatin-1N	26	+3	10.6	0.496	3–10, 12–24
Ocellatin-2N	26	+2	10.2	0.281	12–24
Ocellatin-3N	19	+2	10.2	0.911	4–11, 13–18
Ocellatin-4N	19	+2	10.2	0.874	2–11, 13–18

* Calculations of helicity using the AGADIR program were performed at pH 7, ionic strength 0.1 M, and 278 K.

**Table 2:** Minimum inhibitory concentrations in μM (μg/mL) of synthetic replicates of ocellatins isolated from skin secretions of *L. insularum* and *L. nesiotus* against reference and antibiotic-resistant strains of Gram-positive and Gram-negative bacteria.

*Bacteria*	Ocellatin-1I	Ocellatin-2I	Ocellatin-1N	Ocellatin-3N
**Gram-positive**				
*S. aureus* (ATCC 12600)	250 (575)	>250 (>620)	250 (725)	31.25 (67)
*S. aureus* (ATCC BAA-2312)	250 (575)	>250 (>620)	250 (725)	31.25 (67)
*E. faecium* (ATCC 19434)	n.d.	250 (620)	250 (725)	62.5 (134)
*E. faecalis* (ATCC 51299)	>250 (>575)	>250 (>620)	>250 (>725)	250 (535)
**Gram-negative**				
*E. coli* (ATCC 35218)	62.5 (144)	62.5 (155)	62.5 (181)	31.25 (67)
*K. pneumoniae* (ATCC 49472)	125 (288)	125 (310)	125 (362)	62.5 (134)
*K. pneumoniae* (ATCC BAA-2814) *	>125 (>288)	125 (310)	125 (362)	62.5 (134)
*P. aeruginosa* (ATCC 27853)	n.d.	>125 (>310)	>125 (>362)	62.5 (134)
*S. typhimurium* (ATCC 14028)	250 (575)	125 (310)	250 (725)	62.5 (134)

n.d., not determined. * denotes an antibiotic-resistant strain.
